# Supplementary material for: Insights and protocols for discrimination of sugarcane clones by dissimilarity measures on RGB and NIR data
Source: PLoS One. 2023 Jul 20;18(7):e0288508. doi: 10.1371/journal.pone.0288508 (PMC10359001; doi:10.1371/journal.pone.0288508)

PLOS ONE

Insights and protocols for discrimination of sugarcane clones by dissimilarity measures on RGB and NIR data

--Manuscript Draft--

**Manuscript Number:**	PONE-D-22-33698
**Article Type:**	Research Article
**Full Title:**	Insights and protocols for discrimination of sugarcane clones by dissimilarity measures on RGB and NIR data
**Short Title:**	Discrimination of sugarcane clones by dissimilarity measures
**Corresponding Author:**	Luiz Alexandre peternelli, Ph.D.<br>Universidade Federal de Vicosa<br>Vicosa, Minas Gerais BRAZIL
**Keywords:**	Euclidean distance; Mahalanobis distance; ROC curves; Clone selection; Simplified System
**Abstract:**	In sugarcane breeding, dense experiments have been considered in the initial phase (T1), such as the Simplified System (SS) of genotype selection. In this method, the seedlings of each family are transplanted directly from the seed box to the field, forming a kind of carpet. Despite the practical aspect of the method, selection problems are common, as stalks from the same individual within the family are subject to being taken to later evaluation stages, to the detriment of stalks from different individuals. To facilitate the discrimination of stalks of the same family in SS, we evaluated using RGB images (network:green:blue) and NIR (near infrared) spectra. We applied Euclidean distance (D) and Mahalanobis distance ( ) dissimilarity measures to the image and spectral data to distinguish stalks with different genotypes. RGB and NIR data were taken from type +1 leaf samples collected from two experimental blocks, totaling 31 evaluated families. The analyzes were carried out in two stages. In the first stage, we sought to evaluate the classification capacity using RGB images and NIR spectra, using D as a measure of dissimilarity. In the second step, we developed and validated a protocol using RGB images to classify clones, with as a dissimilarity measure. Preliminary results, with distance D, allowed to discriminate clones based on the distance of the evaluated attributes and their combinations. In addition, with the analyzes using the D distance, it was identified that only the use of the R attribute (red band) would give satisfactory results for the second stage, which was the proposed analysis protocol, applying the distance. The statistic and associated p-value confirmed the protocol's usefulness in discriminating stalks in SS, especially stalks from the same families.
**Order of Authors:**	Luiz Alexandre peternelli, Ph.D.<br>Andrea Carla Bastos Andrade, Ph.D.
**Additional Information:**	
**Question**	**Response**
**Financial Disclosure**<br><br>Enter a financial disclosure statement that describes the sources of funding for the work included in this submission. Review the submission guidelines for detailed requirements. View published research articles from *PLOS ONE* for specific examples.<br><br>This statement is required for submission and **will appear in the published article** if	LAP; (CNPQ) - process 309662/2019-2; www.cnpq.br; The funders had no role in study design, data collection and analysis, decision to publish, or preparation of the manuscript.

the submission is accepted. Please make sure it is accurate.<br><br>**Unfunded studies**<br>Enter: *The author(s) received no specific funding for this work.*<br><br>**Funded studies**<br>Enter a statement with the following details:<br>• Initials of the authors who received each award<br>• Grant numbers awarded to each author<br>• The full name of each funder<br>• URL of each funder website<br>• Did the sponsors or funders play any role in the study design, data collection and analysis, decision to publish, or preparation of the manuscript?<br>• **NO** - Include this sentence at the end of your statement: *The funders had no role in study design, data collection and analysis, decision to publish, or preparation of the manuscript.*<br>• **YES** - Specify the role(s) played.<br><br>* typeset	
**Competing Interests**<br><br>Use the instructions below to enter a competing interest statement for this submission. On behalf of all authors, disclose any competing interests that could be perceived to bias this work—acknowledging all financial support and any other relevant financial or non-financial competing interests.<br><br>This statement is required for submission and **will appear in the published article** if the submission is accepted. Please make sure it is accurate and that any funding sources listed in your Funding Information later in the submission form are also declared in your Financial Disclosure statement.<br><br>View published research articles from *PLOS ONE* for specific examples.	The authors have declared that no competing interests exist.

**NO authors have competing interests**<br><br>Enter: *The authors have declared that no competing interests exist.*<br><br>**Authors with competing interests**<br><br>Enter competing interest details beginning with this statement:<br><br>*I have read the journal's policy and the authors of this manuscript have the following competing interests: [insert competing interests here]*<br><br>* typeset	
**Ethics Statement**<br><br>Enter an ethics statement for this submission. This statement is required if the study involved:<br><br>• Human participants<br>• Human specimens or tissue<br>• Vertebrate animals or cephalopods<br>• Vertebrate embryos or tissues<br>• Field research<br><br>Write "N/A" if the submission does not require an ethics statement.<br><br>General guidance is provided below. Consult the submission guidelines for detailed instructions. **Make sure that all information entered here is included in the Methods section of the manuscript.**	N/A

Powered by Editorial Manager® and ProduXion Manager® from Aries Systems Corporation

**Format for specific study types**<br><br>**Human Subject Research (involving human participants and/or tissue)**<br>• Give the name of the institutional review board or ethics committee that approved the study<br>• Include the approval number and/or a statement indicating approval of this research<br>• Indicate the form of consent obtained (written/oral) or the reason that consent was not obtained (e.g. the data were analyzed anonymously)<br><br>**Animal Research (involving vertebrate animals, embryos or tissues)**<br>• Provide the name of the Institutional Animal Care and Use Committee (IACUC) or other relevant ethics board that reviewed the study protocol, and indicate whether they approved this research or granted a formal waiver of ethical approval<br>• Include an approval number if one was obtained<br>• If the study involved *non-human primates*, add *additional details* about animal welfare and steps taken to ameliorate suffering<br>• If anesthesia, euthanasia, or any kind of animal sacrifice is part of the study, include briefly which substances and/or methods were applied<br><br>**Field Research**<br><br>Include the following details if this study involves the collection of plant, animal, or other materials from a natural setting:<br>• Field permit number<br>• Name of the institution or relevant body that granted permission	
**Data Availability**<br><br>Authors are required to make all data underlying the findings described fully available, without restriction, and from the time of publication. PLOS allows rare exceptions to address legal and ethical concerns. See the PLOS Data Policy and FAQ for detailed information.	Yes - all data are fully available without restriction

Powered by Editorial Manager® and ProduXion Manager® from Aries Systems Corporation

A Data Availability Statement describing where the data can be found is required at submission. Your answers to this question constitute the Data Availability Statement and **will be published in the article**, if accepted.<br><br>**Important:** Stating 'data available on request from the author' is not sufficient. If your data are only available upon request, select 'No' for the first question and explain your exceptional situation in the text box.<br><br>Do the authors confirm that all data underlying the findings described in their manuscript are fully available without restriction?	
**Describe where the data may be found in full sentences. If you are copying our sample text, replace any instances of XXX with the appropriate details.**<br><br>• If the data are **held or will be held in a public repository**, include URLs, accession numbers or DOIs. If this information will only be available after acceptance, indicate this by ticking the box below. For example: *All XXX files are available from the XXX database (accession number(s) XXX, XXX.)*.<br>• If the data are all contained **within the manuscript and/or Supporting Information files**, enter the following: *All relevant data are within the manuscript and its Supporting Information files.*<br>• If neither of these applies but you are able to provide **details of access elsewhere**, with or without limitations, please do so. For example:<br><br>*Data cannot be shared publicly because of [XXX]. Data are available from the XXX Institutional Data Access / Ethics Committee (contact via XXX) for researchers who meet the criteria for access to confidential data.*<br><br>*The data underlying the results presented in the study are available from (include the name of the third party*	The data underlying the results presented in the study are available from Luiz Alexandre Peternelli (peternelli@ufv.br).

and contact information or URL).<br>• This text is appropriate if the data are owned by a third party and authors do not have permission to share the data.<br><br>* typeset	
Additional data availability information:	Tick here if the URLs/accession numbers/DOIs will be available only after acceptance of the manuscript for publication so that we can ensure their inclusion before publication.

Powered by Editorial Manager® and ProduXion Manager® from Aries Systems Corporation

Manuscript

Click here to access/download;Manuscript;2022_12_08 - artigo - compara amostras - ingles.docx

Insights and protocols for discrimination of sugarcane clones by dissimilarity measures on RGB and NIR data

~~**Discrimination of sugarcane clones by dissimilarity measures**~~

Luiz Alexandre Peternelli[1*] **Andréa Carla Bastos Andrade[1]**

[1]Department of Statistics, Federal University of Viçosa, Viçosa, MG, Brazil.

[*]Corresponding author

E-mail: peternelli@ufv.br (LAP)

Abstract

In sugarcane breeding, dense experiments have been considered in the initial phase (T1), such as the Simplified System (SS) of genotype selection. In this method, the seedlings of each family are transplanted directly from the seed box to the field, forming a kind of carpet. Despite the practical aspect of the method, selection problems are common, as stalks from the same individual within the family are subject to being taken to later evaluation stages, to the detriment of stalks from different individuals. To facilitate the discrimination of stalks of the same family in SS, we evaluated using RGB images (~~network~~ red :green:blue) and NIR (near infrared) spectra. We applied Euclidean distance (D) and Mahalanobis distance (D^2) dissimilarity measures to the image and spectral data to distinguish stalks with different genotypes. RGB and NIR data were taken from type +1 leaf samples collected from two experimental blocks, totaling 31 evaluated families. The analyzes were carried out in two stages. In the first stage, we sought to evaluate the classification capacity using RGB images and NIR spectra, using D as a measure

of dissimilarity. In the second step, we developed and validated a protocol using RGB images to classify clones, with D^2 as a dissimilarity measure. Preliminary results, with distance D, allowed to discriminate clones based on the distance of the evaluated attributes and their combinations. In addition, with the analyzes using the D distance, it was identified that only the use of the R attribute (red band) would give satisfactory results for the second stage, which was the proposed analysis protocol, applying the D^2 distance. The D^2 statistic and associated p-value confirmed the protocol's usefulness in discriminating stalks in SS, especially stalks from the same families.

Introduction

The selection of superior genotypes within a sugarcane population is a long-term job, with at least ten years to generate results. On average, one new variety is obtained for every 250 thousand seedlings evaluated in the first stage of the breeding program [1]. Within the genetic breeding programs of this crop, the conventional selection system is the primary way to obtain improved varieties [2]. However, in the conventional method, evaluating a high number of genotypes in the initial stages of breeding is impractical as financial and physical limitations, such as labor and experimental area, may occur [3].

A new genotype selection methodology, called Simplified System (SS) [4], has been evaluated to minimize the difficulties of the conventional system [5]. The method consists of planting in the field densely, or else, all seedlings are transplanted directly from the sowing box to the field, forming a kind of carpet. The genotypes are selected a few months after planting in the field, selecting the individuals with the best vigor [6].

This new selection methodology has been introduced in some of Brazil's sugarcane genetic breeding programs. However, due to the conditions of density in the field caused by this selection system, it may be challenging to select the genotypes precisely without having

identical duplicates when selecting vigorous individuals close to each other and from the same plant. Thus, it is necessary to develop procedures that help differentiate these genotypes in SS.

A possible procedure for the correct identification of different stalks is to carry out the phenotypic evaluation of samples in a non-destructive, fast, accurate, and precise way using digital cameras, sensors, and automatic mechanical devices [7] or using near-infrared (NIR) instruments [8]. In the literature, many studies have evaluated the application of NIR and RGB (Red:Green:Blue) spectroscopy to infer better fruit maturation [9,10], study changes in color and appearance in processed foods [11], evaluate viral infection in plants [12], as well as to differentiate related species [13]. Through these techniques, it is also possible to discriminate genotypes of the same species, as reported in the studies by [14], [15], [16], and [17].

In general, these studies use multivariate analysis techniques for comparison purposes. These analyzes assist in differentiating samples to identify those biologically relevant spectral characteristics [18]. Among the multivariate methods used, such as principal component analysis, discriminant analysis, and clustering, emphasis is placed on the use of dissimilarity measures, such as Euclidean distance and Mahalanobis distance, among others [19]. These statistical approaches applied to NIR and RGB image information can be very valuable in using SS in the initial phase of sugarcane breeding programs, maximizing gains in selection processes.

Given the above, the present work aims to: i) evaluate the potential use of NIR instruments and RGB cameras to distinguish sugarcane stalks as to their origin in the experimental plot; ii) establish a protocol for data collection and a quick and non-destructive analysis that infers about the origin of the stalk samples within densely populated families, which is the case of the SS, that is, if the samples of vigorous stalks that are close within the plot are obtained from genetically different individuals, that is, different clones.

Material and Methods

Plant material

Sugarcane clones were evaluated in an experiment conducted in the Simplified System (SS) of the Sugarcane Genetic Breeding Program of the Federal University of Viçosa (PMGCA-UFV), reported in [5]. In this study, each block had plots containing a particular genotype selected from a given SS family. We collected samples from two experimental blocks for the present study. In block 1, images from 14 clones were obtained, while in block 2, images from 24 clones were obtained. There were 31 families, of which 7 had representatives in both blocks. From each plot containing a single clone, three leaves of the "+1" type (the first leaf wholly detached from the plant sheath) [20,21] were collected, considering those two experimental blocks (Fig 1).

Fig 1. Scheme of the experimental procedure for the origin of samples and data collection. (A): dense planting of seedlings from a given family i (i = 1 to F, the number of families evaluated) in the simplified system; (B) the stalks of the most vigorous individuals in each family were selected and planted in new plots; in this case each, h block contains an individual selected from each family i; (C) three +1 leaves were randomly collected from each plot; (D) from each leaf, RGB and NIR data were obtained.

Table 1 represents the classification form for the set of samples obtained for analysis.

Table 1. Definition of classes referring to pairs of leaf samples used in comparisons according to the plot from which the samples were obtained.

Class	Family	Block	Description
C1	The same	The same	The leaves collected represent samples from the same individual
C2	The same	Different	The leaves collected represent samples from different individuals, but from the same family
C3	Different	The same or different	The collected leaves represent samples of individuals from different families and, therefore, different individuals.

Doesn't leaf coloration change with culture time? Were the samples collected at the same culture day?

Obtaining RGB (Red:Green:Blue) and NIR (near-infrared) data

Leaf samples were collected in the field and identified according~~ly~~ to the family and block of origin. These samples were taken to the laboratory to obtain images and NIR data. First, RGB images were obtained from the middle third of the leaves. For this, a Fujifilm S4000 digital camera with 14 MegaPixels and a 30x Superwide lens was used 20 cm above the leaf. Leaves were previously positioned on a bright plane and between two light reflectors built to homogenize the lighting of the photos obtained (Fig 1D).

Additionally, using the DLP®NIRscan™ Nano EVMO spectrometer, each leaf sample had its NIR spectrum obtained in absorbance mode in an investigated range of wavelengths from 900 to 1700 nm. To obtain the spectra, we followed a sampling model where the blade of the leaves was removed from the midrib and folded so that the instrument could ~~be~~ read (Fig

1D). In this way, the energy fired by the device would not cross the leaf and cause unwanted dispersion.

Before applying the dissimilarity measurement algorithms, the NIR spectrum matrix was subjected to different pre-treatments and their combinations (~~PASQUINI, 2018~~ [18]; ~~GONÇALVES et al., 2021~~ [25]) to verify the best procedure for this type of analysis. Each pre-treatment was tested to verify if the differentiation between the samples would be potentiated after the spectra pre-treatment. In the end, in addition to the original spectrum matrix, the following transformations were tested: Savitzky-Golay Smoothing, First Derivative, Second Derivative, and Multiplicative Scattering Correction (MSC), in addition to the Mean Centering pre-processing [8].

Statistical framework

The analyses were carried out in two stages to achieve this research's objectives.

Initially, we evaluated the discrimination potential of different individuals, or, conversely, the identification of identical individuals, that is, from the exact clone, based on data from RGB images and NIR spectra. Once the possibility of using images or NIR to classify clones was identified (Table 1), the second stage of the research was to develop a quick and practical protocol for this purpose.

Considering that, at the field level, it would be easier to obtain images of the leaves and guarantee the success of step 1, the second step of this research consisted of the development of a protocol for collecting images of two samples of leaves for decision making regarding whether they belong to the same or different individuals. The protocol described in this work was tested on a subset of the images.

Euclidean distance (D) as a measure of dissimilarity

The Euclidean distance [19,22] was used to compare the attributes of pairs of images organized into three distinct classes (Table 1). In the first stage of this research, the leaf attributes were obtained in two ways: RGB and NIR. Additionally, these attributes were aggregated to verify the eventual sum of effects and, therefore, the power of sample discrimination.

please further explain how this parameter was used in this work

The discrimination of samples according to the Euclidean Distance of the attributes obtained based on NIR, RGB and NIR+RGB was evaluated under different scenarios (Table 2). ROC (Receiver Operating Characteristic) curves [23], representing the relationship between false positive and true positive rates, were used for the comparison. The determination of the ROC curve and the area under the curve (AUC - a measure of the discriminatory capacity of the classification algorithm) is related to the construction of the confusion matrix and the calculation of sensitivity and specificity measures [24–26]. It is noteworthy that, in the present study, all attributes were standardized prior to data analysis.

please elaborate more on the need for standardization for the employed metrics (aren't some of them already standardized by default?) and the method employed

Table 2. Scenarios used in classifying samples regarding the corresponding Euclidean distance values obtained between pairs of images or NIR spectra.

Scenarios	Origin of attributes used
RGB	RGB image
R	Red from RGB image
NIR	NIR spectrum after pre-treatment
RGB+NIR	RGB image combined with pre-treated NIR spectrum
R+NIR	Red combined with pre-treated NIR spectrum

RGB, Red, Green, or Blue obtained from digital images; NIR, near-infrared.

Mahalanobis distance (D^2) as a measure of dissimilarity

The Mahalanobis distance [19,22] was used in the work's second step to develop a quick and straightforward protocol for decision-making on classifying pairs of RGB images collected in the field. This protocol would be necessary if we have images of stalks under selection in the SS system that are sufficiently close to each other, which could lead to doubts about whether such clones could come from the same plant.

The proposed protocol is to compare two images using the following procedure:

1) Collect the +1 leaf image of two vigorous stalks located so close together that it may seem suspicious whether or not the stalks belong to the same individual;
2) Take an image from each leaf and use a resampling [23] approach to select a number n of pixels from each image; n is sufficiently large and smaller than the total number of pixels in the image. Each sampling of pixels will compose a new image, called the fake image of the original image;

"fake image" seems odd to me, What about "resample image"?

3) Obtain the average vector of the attributes of each fake image;
4) Calculate the differences between the attributes obtained from pairs of fake images;
5) Obtain the same attributes from each of the two original images;
6) Obtain the average vector of the attributes of each original image;
7) Calculate the difference between the attributes obtained in the previous step;
8) Construct the combined covariance matrix of the attribute differences from steps 4 and 7;
9) Calculate the Mahalanobis distance between the mean vector obtained in step 7 and the vector of the population mean obtained in step 4, weighted by the matrix obtained in 8; the corresponding p-value is evaluated.

Fig 2 outlines the procedure used to aid decision-making regarding the similarity between the two selected stalks.

Fig 2. Protocol used to aid decision-making based on the Mahalanobis distance (D^2), and the corresponding p-value.

(A) RGB images are obtained from two suspicious samples. (B) from each image, a large F-number of "fake" images is obtained from a pixel resampling process of the original images. (C) Attributes are taken from the original images and the resulting "fake" images. (D) we obtain the measure D^2 and the associated p-value.

The rationale presented in this protocol is based on the theory of decomposition (between and within) of the variability between observations of two treatments (or factors) under consideration, the so-called Analysis of Variance (ANOVA) [27,28]. In the case of ANOVA, the difference between the means of two treatments (in our case, two original images) is evaluated by comparing their variability (variability between) with the variability arising from repetitions of the treatments (variability within) using the F test. In our work, we will use the chi-square statistic with p (where p = number of attributes) degrees of freedom for the evaluation of Mahalanobis statistic D^2 [19,22]. The statistic D^2 is used to evaluate the difference between the vector of differences for the attributes of the original images ("between") and the mean vector of the differences for the attributes of the numerous "fake" images obtained from each image ("within"), weighted by the combined covariance matrix of the attributes of all images.

To obtain the p-value associated with the D^2 statistic, we need to assume that the population under consideration has a multivariate normal distribution so that the values of $D^2 \sim \chi^2(p)$ [22].

have the authors determined (by an appropriate statistical method) if this assumption is correct?

Computational resources

The analysis was carried out with the R software [29]. Additional R functions, when necessary, were developed at the Laboratory of Analysis and Research in Applied Statistics (LAPEA, www.lapea.ufv.br).

Results and Discussion

As commented above, this work had two stages. The first was to assess the possibility of distinguishing between samples from the exact clone or different clones, and the second defined the investigation protocol on pairs of RGB images of suspicious samples.

Step 1

were the three images (R, G, and B) analysed individually or was a combination of the 3 used in a single image used?

At this stage of the work, we evaluated using RGB images, NIR spectra, and the combination of both as tools for the discrimination of sugarcane clones.

Initially, we evaluated the use of combined R, G, and B bands (Fig 3A), only R (Fig 3B), and only NIR (Fig 3C) in the discrimination of images previously classified as class C1 (samples from the exact clone) and their complement, that is, all results from classes C2 and C3 (samples from different clones, whether from the same family or not). In this preliminary analysis, it can be seen that there is a concentration of lower values of Euclidean distances when the samples come from the exact clone (as can be seen by observing the median value in each boxplot), as would be expected. Such results are indicators that it would be possible, in practice, to find a way to discriminate clones based on the Euclidean distance of these attributes, as used elsewhere in other applications [14,15,17,30,31].

please elaborate more. what were the used wavelengths (all, solely a few selected wavelengths, etc.)?

Fig 3. Distribution of Euclidean distances (D) for pairs of C1 (green) and non-C1 (orange) class samples.

(A) Use R, G, and B bands as attributes obtained from leaf images. (B) use only the R band as an attribute of these images. (C) use of attributes corresponding to the wavelengths of the pre-treated NIR spectra of leaf samples. The boxplots inserted in the graphs are helpful for better interpreting the descriptive measures, such as the median, of the D.

It is essential to inform that the identification of differences between the samples was improved after the application of pre-treatment on the NIR spectra matrix, possibly due to the increase in the signal-to-noise ratio after the application of certain pre-treatments [32,33]. In the current data set, the best results occurred after the Savitzky-Golay Smoothing (with window size 5 and degree 2 polynomial), First Derivative, and Multiplicative Scatter Correction (MSC) transformations, followed by Mean Centering pre-processing method. These pre-treatments are very common in sugarcane works [33–36].

To better compare the different alternatives of the sample pairs classification process, we constructed graphs (Fig 4) showing the ROC curves corresponding to the scenarios defined in Table 2.

Fig 4. ROC curves for clone differentiation procedures based on RGB images and near-infrared (NIR) spectra.

(A) use of attributes R, G, and B (black line), NIR (green), and the combination of RGB and NIR (blue line). (B) use only the attribute R (black line), NIR (green), and the combination of R and NIR (blue line). It is essential to mention that the green and blue lines are superimposed because they present similar results. TPR: true positive rate. FPR: false positive rate.

doesn´t the blue line in graph 4A represents "all bands (NIR+RGB) together"? And if so, how can the AUC of that line be so close to the AUC of the R band (black line on 4B? They seem quite apart analyzing figure 4A and 4B.

The ROC curves of the different procedures (Fig 4) indicate their potential for discriminating pairs of samples since such curves are above the 45-degree line corresponding to random classification [23]. It is also observed that using only the R band (AUC = area under the curve = 0.6219) offers practically the same power of discrimination as all bands together (AUC = 0.6289). On the other hand, the greatest discriminatory power would occur using attributes obtained from the NIR (AUC = 0.7348) data. Furthermore, the concatenation of NIR and RGB data (AUC = 0.7360) or NIR and R (AUC = 0.7352) did not significantly improve the clone discrimination process, probably because there are many more attributes from the NIR spectra (605 wavelengths) compared to the attributes derived from the images. Because NIR instruments capture information at the molecular level in leaves [8] and not just color variations in the visible spectrum, better performance was expected from using attributes in NIR.

In all evaluated scenarios (Fig 4), the ROC curves indicate that the best results occurred for the NIR after pre-treatment of the spectrum matrix. However, given the difficulty of having portable instruments in breeding programs for the rapid collection of spectra, in addition to the need to evaluate different pre-treatments in the spectra matrix, which can be a difficulty in the analysis, the use of NIR data passes not to be a good option for use in practice.

Thus, from a practical point of view, the easiest way would be to collect RGB images at the field level, which justifies the development of an image collection and analysis protocol for stalks discrimination in the initial stages of the SS.

Step 2

At this stage of the work, we used only the RGB images to evaluate the second objective of this research. In particular, considering the previous analysis already discussed in the

previous item, it was identified that only the use of the R attribute would give satisfactory results.

D^2 values and corresponding p-values were obtained from the analysis of 2601 image combinations between pairs of samples from seven families that presented genotypes in the two experimental blocks under study. Of these 2601 pair combinations, there were 36 from class C1, 63 from class C2, and 2502 from class C3, as defined in Table 1.

Considering the asymmetry obtained between the D^2 values , and between the p-values of the image pairs, we chose to calculate the median of these values in the observed data. For D^2, the median of class C1 was the lowest (0.8149) when compared to the medians of classes C2 (2.6163) and C3 (3.5948). As expected, the median pvalue for class C1 (0.3744) was higher than that of the other classes (0.1057 and 0.0579 for classes C2 and C3, respectively). These results confirm the usefulness of the proposed protocol (Fig 2) for genotype discrimination.

On the other hand, comparing individuals from different families is not that important in practice since, in the SS, the families are planted far from each other [5]. The biggest problem is differentiating clones within the same family in this SS. It can happen that two vigorous individuals within the same family, and which would therefore be selected, are so close to each other that we would be in doubt whether they are shoots of the same plant, that is, a single individual, or whether in fact, they are distinct individuals. To assess this issue, we compared the results for individuals in class C1 against those in class C2 (Fig 5).

both these values are higher than 0.05. Doesn't that infer that the majority of the attributes from individuals from the same family (C2) and from different families (C3) are not statistically different?

Fig 5. Distribution of Mahalanobis distance (D^2) and p-values for comparing the differences between samples' attributes.

Class C1 (orange): The leaves collected represent samples from the same individual. Class C2 (green): The collected leaves represent samples from individuals from the same family.

shouldn't that be 0.05 (for 95% significance)?

Considering that the value of D^2 is affected by the number of attributes in the data matrix, a future investigation would try to identify the p-value threshold that would separate the images of different leaves of the same individual from the rest. If it is impossible to find this threshold, at least the researcher can decide the similarity of the compared stalks. The argument will be that if the images are from the same genotype, collected from different leaves, the distance D^2 should be zero. Logically, it would never be zero, as the images would never be identical, but a certain threshold h would make the probability of concluding that two images correspond to the exact clone occur if the distance is smaller than h. In our work, we did not try to find a threshold value h for D^2, since this value can be influenced by the number of attributes extracted from the images. However, we will consider the p-value associated with the calculated D^2 as an additional measure for decision-making. If the p-value associated with the D^2 obtained from the comparison of the two images is large (and, therefore, with a relatively small D^2), we would indicate that the images are similar and with a high probability of belonging to the same individual. On the other hand, if the p-value is small, there is an indication that the images were obtained from different individuals .

Conclusions

The Euclidean distance of the attributes derived from RGB images and NIR spectra allows for the discrimination of stalks suspected of being parts of the same individual within the family being evaluated in the Simplified Selection System. The distinction is made more efficient by using NIR spectra. However, as it is more practical in the field, RGB images would be preferred. The proposed protocol for the inference about any two images proved to be efficient in classifying them as to whether they belong to the same individual and is, therefore, useful in choosing stalks in the simplified selection system.

Acknowledgments

The authors would like to thank the Interuniversity Network for the Development of the Sugarcane Industry (RIDESA) for providing the data and field services carried out in the UFV's sugarcane breeding program, the National Council for Scientific and Technological Development (CNPQ) and FINEP for the research grants.

References

1. Dal-Bianco M, Carneiro MS, Hotta CT, Chapola RG, Hoffmann HP, Garcia AAF, et al. Sugarcane improvement: How far can we go? Current Opinion in Biotechnology. 2012. pp. 265–270. doi:10.1016/j.copbio.2011.09.002
2. de Morais LK, de Aguiar MS, de Albuquerque E Silva P, Marinho Câmara TM, Cursi DE, Fernandes Jú Nior AR, et al. Breeding of sugarcane. Industrial Crops: Breeding for Bioenergy and Bioproducts. Springer New York; 2015. pp. 29–42. doi:10.1007/978-1-4939-1447-0_2
3. Peternelli LA, Souza EFM de, Barbosa MHP, Carvalho MP de. Delineamentos aumentados no melhoramento de plantas em condições de restrições de recursos. Ciência Rural. 2009;39: 2425–2430. doi:10.1590/S0103-84782009005000209
4. Melo LJOT. Sistema simplificado de seleção para a fase inicial do melhoramento genético da cana-de-açúcar. Tese, Universidade Federal do Paraná. 2014. Available: https://acervodigital.ufpr.br/handle/1884/36375
5. Ferreira PHS, Gonçalves MTV, Teixeira G, Ferreira M de P, de Oliveira RL, Barbosa MHP, et al. Comparison of family selection methodologies used in the initial phase of sugarcane breeding. Crop Sci. 2022;62: 679–689. doi:10.1002/csc2.20685
6. Daros E, Zambon JLC, Oliveira RA. Programa de melhoramento Genético da Cana-de-Açúcar da UFPR: 25 anos de pesquisa. 1 ed. Curitiba: Graciosa; 2016.
7. Großkinsky DK, Svensgaard J, Christensen S, Roitsch T. Plant phenomics and the need for physiological phenotyping across scales to narrow the genotype-to-phenotype knowledge gap. Journal of Experimental Botany. Oxford University Press; 2015. pp. 5429–5440. doi:10.1093/jxb/erv345
8. Pasquini C. Near infrared spectroscopy: A mature analytical technique with new perspectives – A review. Analytica Chimica Acta. Elsevier B.V.; 2018. pp. 8–36. doi:10.1016/j.aca.2018.04.004

9. Menesatti P, Zanella A, D'Andrea S, Costa C, Paglia G, Pallottino F. Supervised multivariate analysis of hyper-spectral NIR images to evaluate the starch index of apples. Food Bioproc Tech. 2009;2: 308–314. doi:10.1007/s11947-008-0120-8

10. Li B, Lecourt J, Bishop G. Advances in non-destructive early assessment of fruit ripeness towards defining optimal time of harvest and yield prediction—a review. Plants. MDPI AG; 2018. doi:10.3390/plants7010003

11. Fongaro L, Alamprese C, Casiraghi E. Ripening of salami: Assessment of colour and aspect evolution using image analysis and multivariate image analysis. Meat Sci. 2015;101: 73–77. doi:10.1016/j.meatsci.2014.11.005

12. Haq QMI, Mabood F, Naureen Z, Al-Harrasi A, Gilani SA, Hussain J, et al. Application of reflectance spectroscopies (FTIR-ATR & FT-NIR) coupled with multivariate methods for robust in vivo detection of begomovirus infection in papaya leaves. Spectrochim Acta A Mol Biomol Spectrosc. 2018;198: 27–32. doi:10.1016/j.saa.2018.02.065

13. Shawky E, Selim DA. NIR spectroscopy-multivariate analysis for discrimination and bioactive compounds prediction of different Citrus species peels. Spectrochim Acta A Mol Biomol Spectrosc. 2019;219: 1–7. doi:10.1016/j.saa.2019.04.026

14. Kim SW, Min SR, Kim J, Park SK, Kim T il, Liu JR. Rapid discrimination of commercial strawberry cultivars using Fourier transform infrared spectroscopy data combined by multivariate analysis. Plant Biotechnol Rep. 2009;3: 87–93. doi:10.1007/s11816-008-0078-z

15. Cui HF, Ye ZH, Xu L, Fu XS, Fan CW, Yu XP. Automatic and rapid discrimination of cotton genotypes by near infrared spectroscopy and chemometrics. J Anal Methods Chem. 2012;1. doi:10.1155/2012/793468

16. Feng X, Peng C, Chen Y, Liu X, Feng X, He Y. Discrimination of CRISPR/Cas9-induced mutants of rice seeds using near-infrared hyperspectral imaging. Sci Rep. 2017;7. doi:10.1038/s41598-017-16254-z

17. Alves Filho, Silva, Lima, Ribeiro, Silva, Zocolo, et al. Metabolomic Variability of Different Genotypes of Cashew by LC-Ms and Correlation with Near-Infrared Spectroscopy as a Tool for Fast Phenotyping. Metabolites. 2019;9: 121. doi:10.3390/metabo9060121

18. Liland KH. Multivariate methods in metabolomics - from pre-processing to dimension reduction and statistical analysis. TrAC - Trends in Analytical Chemistry. 2011. pp. 827–841. doi:10.1016/j.trac.2011.02.007

19. Khatree R, Naik DN. Multivariate data reduction and discrimination with SAS software. New York: John Wiley and Sons; 2000.

remove ALL CAPS

20. MOORE PH, TEW TL. Morphology and Anatomy. In: HEINZ DJ, editor. Sugarcane improvement through breeding. Amsterdam: Elsevier; 1987. pp. 85–142.

21. Porto N de A, Roque J v., Wartha CA, Cardoso W, Peternelli LA, Barbosa MHP, et al. Early prediction of sugarcane genotypes susceptible and resistant to Diatraea saccharalis using spectroscopies and classification techniques. Spectrochim Acta A Mol Biomol Spectrosc. 2019;218: 69–75. doi:10.1016/j.saa.2019.03.114

22. Manly BFJ. Métodos estatísticos multivariados: uma introdução. 3rd ed. Porto Alegre: Bookman; 2008.

23. James G, Witten D, Hastie T, Tibshirani R. An Introduction to Statistical Learning. New York: Springer US; 2013.

24. Gajowniczek K, Ząbkowski T, Szupiluk R. Estimating the ROC Curve and its significance for classification models' assessment. QUANTITATIVE METHODS IN ECONOMICS. 2014.

remove ALL CAPS

25. Gonçalves L, Subtil A, Oliveira MR, De P, Bermudez Z. ROC curve estimation: an overview. REVSTAT-Statistical Journal. 2014.

26. Jiménez-Valverde A. Insights into the area under the receiver operating characteristic curve (AUC) as a discrimination measure in species distribution modelling. Global Ecology and Biogeography. 2012;21: 498–507. doi:10.1111/j.1466-8238.2011.00683.x

27. Steel RGD, Torrie JH, Dickey DA. Principles and procedures of statistics: A biometrical approach. 2nd ed. Tokyo: Mcgraw-Hill Kogakusha; 1980.

28. Bowerman BL, O'Connell RT. Linear statistical models: An applied approach. Belmont: Duxbury; 1990.

29. R Core Team. R: A language and environment for statistical computing. Vienna: R Foundation for Statistical Computing; 2021.

30. Ribera J, He F, Chen Y, Habib AF, Delp EJ. Estimating Phenotypic Traits From UAV Based RGB Imagery. 2018. Available: http://arxiv.org/abs/1807.00498

31. Brambilla M, Romano E, Buccheri M, Cutini M, Toscano P, Cacini S, et al. Application of a low-cost RGB sensor to detect basil (Ocimum basilicum L.) nutritional status at pilot scale level. Precis Agric. 2021;22: 734–753. doi:10.1007/s11119-020-09752-0

32. Liu Y, Liu Y, Chen Y, Zhang Y, Shi T, Wang J, et al. The influence of spectral pre-treatment on the selection of representative calibration samples for soil organic matter estimation using vis-NIR reflectance spectroscopy. Remote Sens (Basel). 2019;11. doi:10.3390/rs11040450

33. Peternelli LA, Gonçalves MTV, Fernandes JG, Brasileiro BP, Teófilo RF. Selection of sugarcane clones via multivariate models using near-infrared (NIR) spectroscopy data. Aust J Crop Sci. 2020;14: 889–896. doi:10.21475/ajcs.20.14.06.p2099

34. Corrêdo L de P, Maldaner LF, Bazame HC, Molin JP. Evaluation of minimum preparation sampling strategies for sugarcane quality prediction by vis-nir spectroscopy. Sensors. 2021;21: 1–23. doi:10.3390/s21062195

35. Gonçalves MTV, Morota G, de Almeida Costa PM, Vidigal PMP, Barbosa MHP, Peternelli LA. Near-infrared spectroscopy outperforms genomics for predicting sugarcane feedstock quality traits. PLoS One. 2021;16. doi:10.1371/journal.pone.0236853

36. Kamwilaisak K, Jutakridsada P, Iamamornphanth W, Saengprachatanarug K, Kasemsiri P, Konyai S, et al. Estimation of sugar content in sugarcane (Saccharum spp.) Variety lumpang 92-11 (LK 92-11) and khon kaen 3 (KK 3) by near infrared spectroscopy. Engineering Journal. 2021;25: 69–83. doi:10.4186/ej.2021.25.3.69

Figure

Click here to access/download;Figure;Fig1.tif

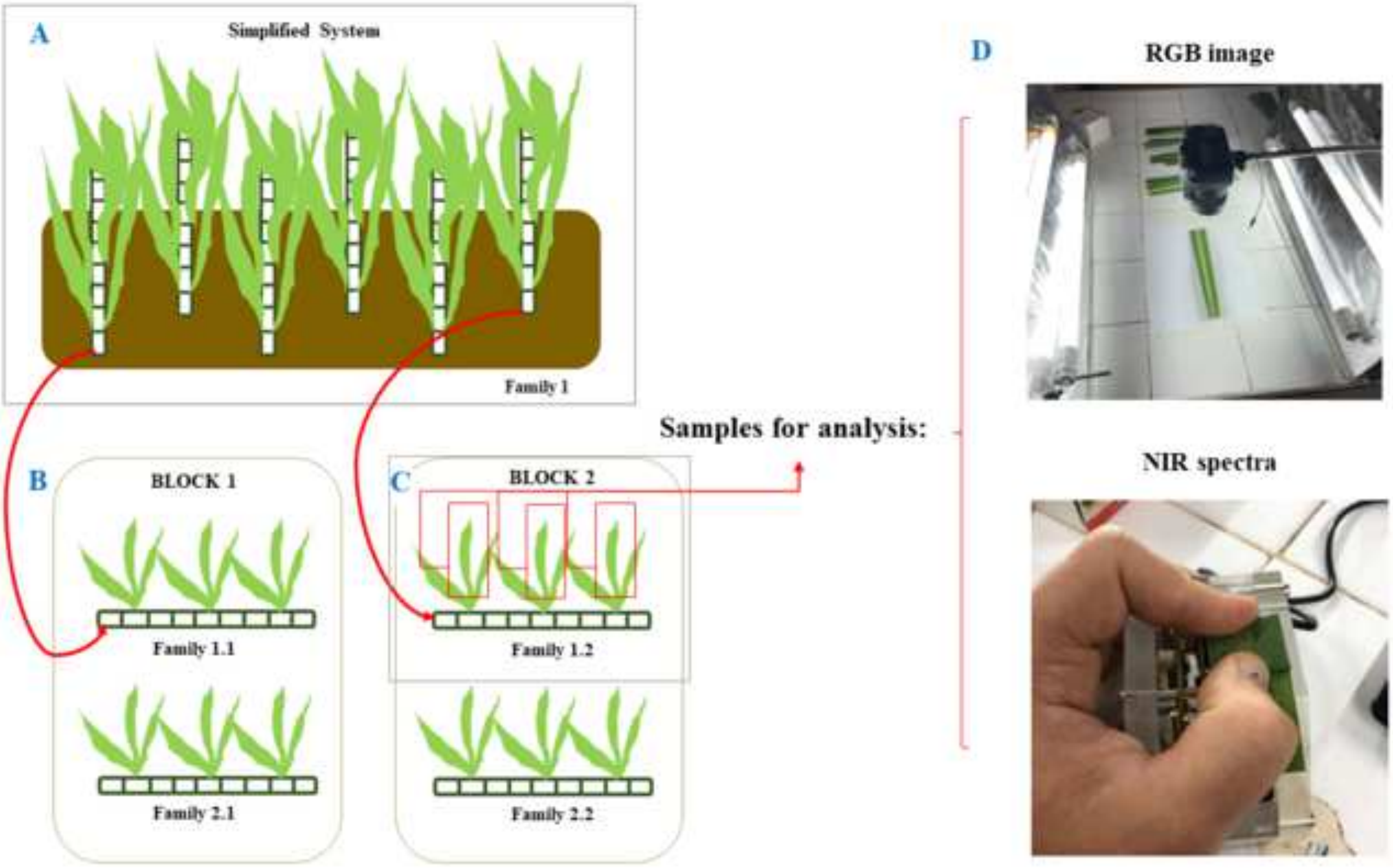

Figure
Click here to access/download;Figure;Fig2.tif

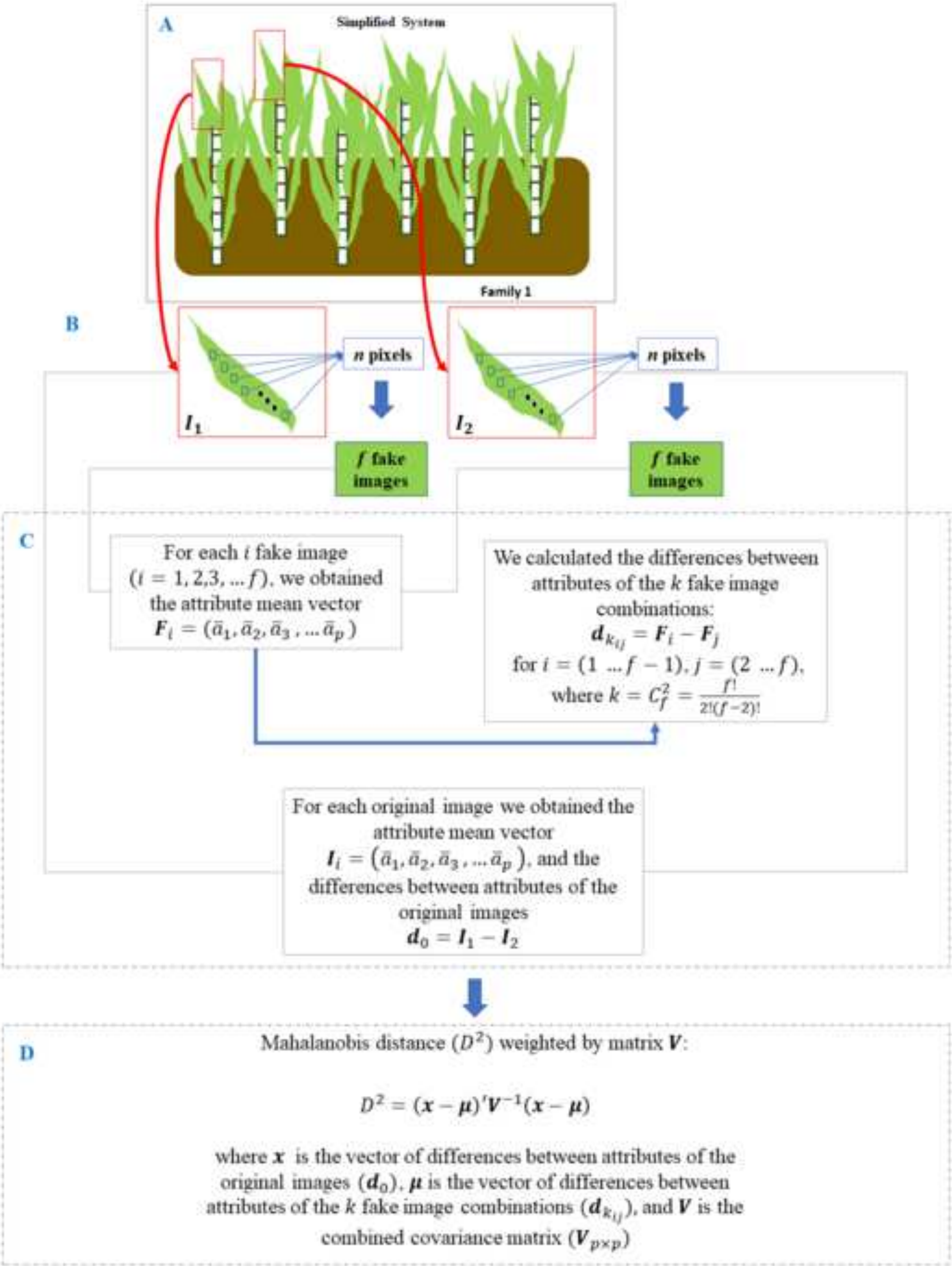

Figure

Click here to access/download;Figure;Fig3.tif

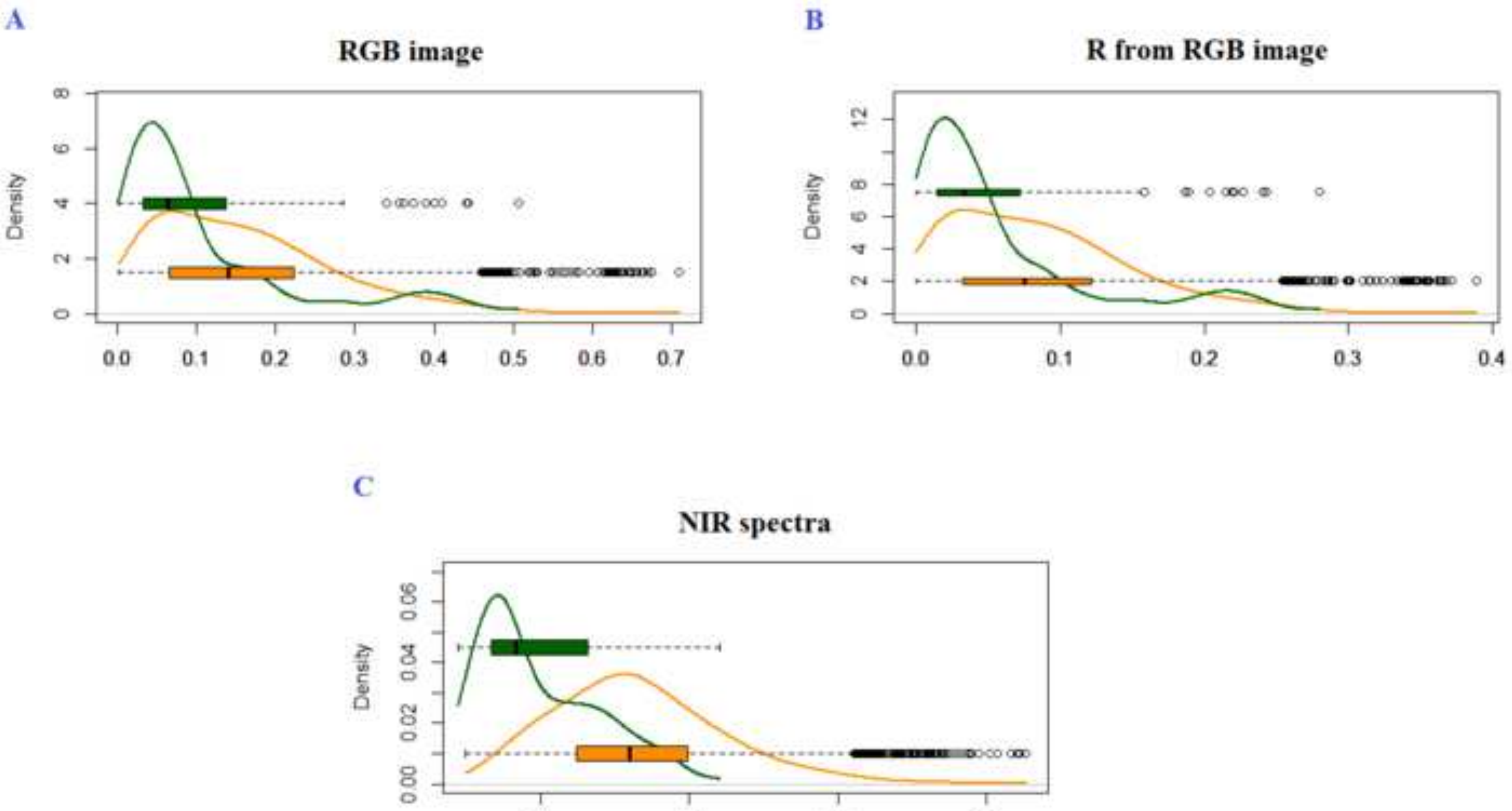

Figure

Click here to access/download;Figure;Fig4.tif

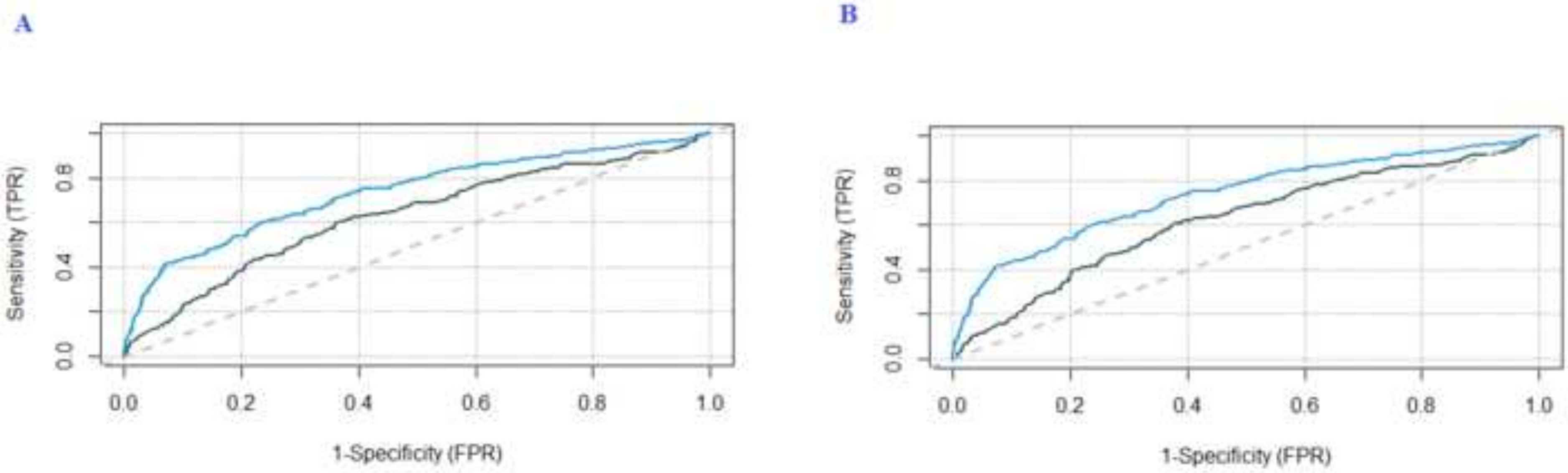

Figure

Click here to access/download;Figure;Fig5.tif

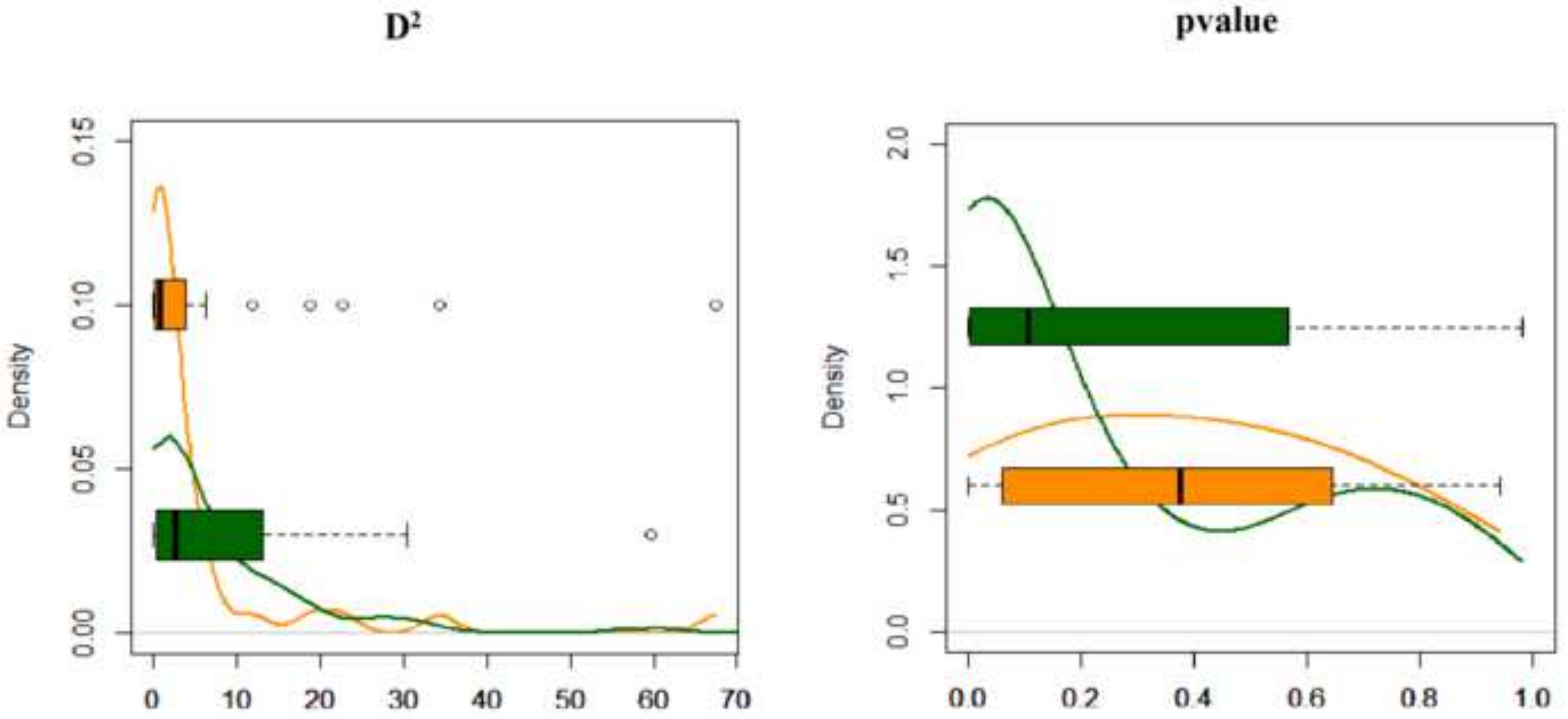

Supplement: Attachment — Submitted filename: PONE-D-22-33698_reviewer-corr.pdf [file pone.0288508.s001.pdf]
