# Supplementary material for: Insights and protocols for discrimination of sugarcane clones by dissimilarity measures on RGB and NIR data
Source: PLoS One. 2023 Jul 20;18(7):e0288508. doi: 10.1371/journal.pone.0288508 (PMC10359001; doi:10.1371/journal.pone.0288508)
Supplement: Attachment — Submitted filename: Response to Reviewers.docx [file pone.0288508.s002.docx]

**Rebuttal letter to the academic editor and reviewers**

We want to thank all for the suggestions, comments, and criticisms regarding our manuscript. Your contributions will, for sure, improve the manuscript. Below you can find, in red, the authors’ responses for each question or comment. The highlighted changes were made to the ‘Revised Manuscript with Track Changes’ file.

**Journal Requirements:**

1. Please ensure that your manuscript meets PLOS ONE's style requirements, including those for file naming. The PLOS ONE style templates can be found at 
<https://journals.plos.org/plosone/s/file?id=wjVg/PLOSOne_formatting_sample_main_body.pdf> and <https://journals.plos.org/plosone/s/file?id=ba62/PLOSOne_formatting_sample_title_authors_affiliations.pdf>.

2. Please expand the acronym “CNPq” as indicated in your financial disclosure so that it states the name of your funders in full.

This information should be included in your cover letter; we will change the online submission form on your behalf.

3. We note that you have indicated that data from this study are available upon request. PLOS only allows data to be available upon request if there are legal or ethical restrictions on sharing data publicly. For more information on unacceptable data access restrictions, please see <http://journals.plos.org/plosone/s/data-availability#loc-unacceptable-data-access-restrictions>.

In your revised cover letter, please address the following prompts:

a) If there are ethical or legal restrictions on sharing a de-identified data set, please explain them in detail (e.g., data contain potentially sensitive information, data are owned by a third-party organization, etc.) and who has imposed them (e.g., an ethics committee). Please also provide contact information for a data access committee, ethics committee, or other institutional body to which data requests may be sent.

b) If there are no restrictions, please upload the minimal anonymized data set necessary to replicate your study findings as either Supporting Information files or to a stable, public repository and provide us with the relevant URLs, DOIs, or accession numbers. For a list of acceptable repositories, please see <http://journals.plos.org/plosone/s/data-availability#loc-recommended-repositories>.

We will update your Data Availability statement on your behalf to reflect the information you provide.

4. We note that you have stated that you will provide repository information for your data at acceptance. Should your manuscript be accepted for publication, we will hold it until you provide the relevant accession numbers or DOIs necessary to access your data. If you wish to make changes to your Data Availability statement, please describe these changes in your cover letter and we will update your Data Availability statement to reflect the information you provide.

**Review Comments to the Author:**

*Reviewer #1:*

i. Original article with an excellent method of contributing to genetic improvement, thinking about large-scale phenotyping strategies. Strategies using RGB images can facilitate the use of these methodologies, when obtained with good calibrations.

**Authors:** Thank you for your consideration regarding the potential contribution of our manuscript.

*Reviewer #2:*

Material and methods

ii. It is not clear the number of samples analyzed. It seems that 14 and 24 clones were analyzed, which is a small number from the agricultural point of view. Extracting more pixels could be sufficient from the computer science point of view, but the authors must clarify this information.

**Authors:** Considering the sample as a clone, 38 samples were analyzed. Each sample contained images of three sheets, totaling 114 images. We believe that these 114 images would be sufficient for this work, that is, to discriminate individuals based on dissimilarity measures and that meet the classes defined in Table 1. Considering that the algorithm worked for a smaller sample size, we concluded it was efficient for our purpose.

iii. The authors should provide more details about the methods, so that is may allow other researchers to perform the experiments or move a step further and not use them if that is the case. Hence, in the next paragraphs I list some details that must be presented:
     a. Regarding the image acquisition procedure, please present further details about the illumination and configuration of the camera (ISO, opening, etc).

**Authors:** In fact, it is essential to have as much information as possible so that any other researcher can reproduce the work. In our case, we ensured that the lighting was the same for obtaining the images, as specified in line 106 of the Material and Methods (original manuscript). Since the camera-to-object distance conditions were constant, we used the digital camera's automatic option. We included this information in the text (lines 109 and 110).

b. Please either add some images in the supplementary Material, or report how the region of interest was extracted, as by figure 1 the image contained sample and background. Was it performed manually or automatically using an algorithm?

**Authors:** We can report how the segmentation was performed to obtain the interest region of the image. We used segmentation from package ‘pliman’. We included this information in the text (lines 110 and 111).

c. Lines 115=116: Please mention the pre-treatment methods used and combinations.

**Authors:** The pre-treatments used in the rows of the spectrum matrix were informed, with only the combinations remaining to be informed (lines 119 to 121, original manuscript). Considering that in this type of work involving NIR, not all the combinations used are specified, we present only the one that gave the best result for our dataset. This information appears in lines 236 to 239 of the results and discussion (original manuscript). As a way of helping the researcher to identify the pre-treatment combinations we used, we added this information in the Material and Methods (lines 126 to 128).

d. Line 164: how many resampled images were used?

**Authors:** On line 164, the number of resampled images is not specified (what we call fake images) since, at this point, we are presenting only the algorithm. In our case, after testing different numbers of fake images (varying from 50 to 1,000), we decided to use 100 fake images. We apologize for not including this information in the results and discussion section. We include this information now in the section Results and Discussion (Step 2), in lines 295 to 297. Notice that we replaced the expression “fake images” with “pixel-resampling images,” as suggested by another reviewer.

e. In line 278, the authors mention 2601 combinations of image resampling. Is this the same number of samples used for NIR statistical analysis? How the authors compared the number of samples for each method? What about when the methods were combined?

**Authors:** There are not 2601 combinations, but 861 combinations corresponding to the samples of the seven families that presented genotypes in two experimental blocks. After rereading this section, we noticed and corrected this error in the manuscript. Answering your question, if the samples match the same number of samples used for NIR, the answer is no. We evaluated using NIR in the first part of the manuscript (Step 1). In this case, we considered more samples, as presented in the item 'Plant material' of Material and Methods (lines 81 to 84 of the original manuscript). In Step 1, 114 samples (38 clones x 3 leaves sampled per clone) were collected. Therefore, 6441 pairs of samples were tested. In the case of Step 2 of the objectives, we selected only those families with representatives in both blocks, as mentioned earlier. They totaled 861 combinations (14 clones x 3 sampled leaves, taken two by two).

Results and discussion

i. Perhaps the authors could present the confusion matrix for the samples classified, in addition to f-score, recall, as it may provide further support for data interpretation by readers, in addition to the ROC curve.

**Authors:** There is no way to create a single confusion matrix, as 6,441 pairs of samples were evaluated. Furthermore, there was no optimal Euclidean Distance threshold for discriminating between samples from different individuals. Therefore, we used ROC curves to assess the discriminatory power of using images, NIR, or a combination of both.

ii. The spectra of samples should be presented, and the main wavelengths influenced by the samples, related to the organic bonds, should be discussed. This could provide a deeper interpretation of the influence of samples in the NIR spectra, and also further support for future research.

**Authors:** If the objective were to use the NIR in predicting some attribute of agronomic interest (for example, fiber content, sucrose content), the association between wavelengths and organic bonds would be interesting. Our research group is developing future studies involving this type of investigation. However, for the present manuscript, our main objective was to evaluate sample discrimination between individuals only. Therefore, we did not state any discussion regarding that matter.

iii. I hereby suggest a few works that may aid the authors in this discussion as they used both image analysis and NIR spectroscopy for similar tasks, but the authors may feel free to find others if suitable:
     a. Computer vision system and near-infrared spectroscopy for identification and classification of chicken with wooden breast, and physicochemical and technological characterization - <https://doi.org/10.1016/j.infrared.2018.11.036>
     b. Assessment of beer quality based on foamability and chemical composition using computer vision algorithms, near infrared spectroscopy and machine learning algorithms - <https://doi.org/10.1002/jsfa.8506>
     c. Automated grapevine cultivar classification based on machine learning using leaf morpho-colorimetry, fractal dimension and near-infrared spectroscopy parameters - <https://doi.org/10.1016/j.compag.2018.06.035>

**Authors:** We appreciate the reading suggestions. They present relevant contributions to research with NIR and in different applications. In general, the studies bring insights into prediction and classification through the interpretation of wavelengths concerning the quality of food and animal and plant origin products, as well as genotypic differentiation of agronomic cultivars. However, as previously mentioned, the primary aim of our research is only to differentiate genotypes and not to associate them with a specific trait. This topic is another front of study under development by our research group. The suggested papers will undoubtedly contribute to future ideas and discussions.

iv. Similarly, please discuss how the channel red may have contributed to identifying the desired samples. Please present some grayscale images in this channel for readers information, and how the wavelength in the visible range may identify the samples.

**Authors:** The entire selection process was done independently and through trial and error. When testing R alone, we found that the results were better than combining the three colors or using G and B alone. This result somehow agrees with the literature, which mentions that the blue color contributes little to the vegetation study. For this reason, some camera manufacturers give the options of changing band B to NIR on their devices (see for example: https://www.mapir.camera/en-gb/pages/survey3-cameras#specs).

Minor comments

The text should be fully revised for academic writing. Several sentences must be corrected, along with some typos. I hereby list a few of them, although there are others:

I suggest the authors to verify in the agricultural field, either with some researcher or other papers, the proper term for sugarcane stalk, as I believe that ‘stem’ is more commonly used.

Line 20: I believe RGB stands for red, green blue. Please check ‘network’

Line 36: I believe that ‘task’ is more appropriate than ‘job’ in this context

Line 111: ‘the instrument could be read’ or the instrument could acquire the spectral information’?

Line 112: instead of energy fired, please use the light emitted

Line 268: what does ‘passes’ mean? Please rephrase the sentence to clarify the information

**Authors:** Thanks for the suggestions and pinpoint corrections. We made these corrections to the text, where they appear. We also rerun a new writing revision to the whole manuscript. For the first suggestion, regarding using the term 'stalk' versus 'stem,' we prefer the term 'stalk' as they appear in the majority of the literature on sugarcane.

*Reviewer #3:*

i. The manuscript “Insights and protocols for discrimination of sugarcane clones by dissimilarity measures on RGB and NIR data” has several scientific findings:
   a. In all the evaluated scenarios R, G, and B (black line), NIR (green), and the combination of RGB and NIR (blue line), the ROC curves indicate that the best results occurred for the NIR after pre-treatment of the spectrum matrix.

However, the Authors have concluded that with the difficulty of having portable instruments in breeding programs for the rapid collection of spectra, in addition to the need to evaluate different pre-treatments in the spectra matrix, which can be a difficult in the analysis, the use of NIR data passes not to be a good option for use in practice.

This is an abrupt conclusion without any supporting evidence. Although, classifiers are able to distinguish all classes, the AUC value for RGB images (R band (AUC = 0.6219) RGB together (AUC = 0.6289) are lower than NIR (AUC = 0.7348) and NIR and RGB data (AUC = 0.7360). Hence, a classifier with NIR and RGB data would be better to utilize.

Only the use of the R attribute would give more satisfactory results than RGB together.

**Authors:** We appreciate the considerations presented concerning our scientific findings. Our research had two distinct objectives. The results indicated the possibility of discrimination of individuals at the field level and that the NIR+RGB combination would be better, as informed in the conclusion of the work (objective 1). For practicality and our own experience, we consider the approach using only RGB images more appropriate for any breeding program (objective 2). The reason is that it would not require a specific instrument for collecting NIR spectra in the field, and also a need to have an expert running the NIR analysis and interpretation, which is much more challenging to be performed.

ii. The analysis of 2601 image combinations between pairs of samples from seven families classified 36 images from class C1, 63 from class C2, and 2502 from class C3 (represent samples of individuals from different families and, therefore, different individuals based on〖 D〗^2 values and corresponding p-values. Which can be differentiated by the traditional ways too.

Although, the objective of the present study is to differentiate clones (C2- represent samples from different individuals, but from the same family) within the same family in this SS, the present study suggests that this class C2 has very low representations in terms of the number of images in total of 2601 images.

**Authors:** We used only the seven families with representatives in two experimental blocks to verify our genotype discrimination procedure. We needed samples meeting the three classes of genotypes, as shown in Table 1, just to be able to check for all these possibilities. However, we did consider only the individuals from classes C1 and C2 since they represent the field's most challenging situation. This information appears on lines 304 to 310 within the Results and Discussion section.

iii. The legend of Fig. 3 is not clear, please elaborate it.

**Authors:** We have included more information on the legend of Fig. 3.

iv. Please explain about outliers in each fig 3 and 5.

**Authors:** the points appearing on the plot do not necessarily are outliers. They are just extreme points that may appear accordingly to the underline distribution of the statistic we depict on the plot. Important to mention that the orange boxplot in Fig. 3 is a mixture of C2 and C3 classes. Of course, some of these points could be outliers for some distribution depending on other factors affecting the samples, like disease spots and nutritional aspects of the leaves. We have included this discussion in the text (lines 311 to 315).

*Reviewer #4*:

A reviewer attachment file is provided with the suggested corrections and modifications to the proposed manuscript. Mainly the authors should focus on: need and method for standardization of the employed metrics; normal distribution assumption correctness; in depth method description and results; in depth analysis of the obtained p-values.

i. Line 103: Doesn’t leaf coloration change with culture time? Were the samples collected at the same culture day?

**Authors:** In fact, leaf coloration may change with crop time. The leaves should be vigorous, healthy, and green. We lack this information in the manuscript. To better explain this detail, we included more information on lines 104 and 105, explaining that the samples were collected in the same week.

ii. Line 146 (area under the curve): Please further explain how this parameter was used in this work.

**Authors:** Thanks for pointing out this aspect of the AUC parameter. We have included a further explanation on lines 156 and 157.

iii. Line 149 (all attributes were standardized): Please elaborate more on the need for standardization for the employed metrics (aren’t some of them already standardized by default?) and the method employed.

**Authors:** We included further explanation regarding the standardization used in our paper and a literature where the reader could achieve extra information. This information appears in lines 158 and 161.

iv. Line 168 (fake): “Fake image” seems odd to me, what about “resample image”? **Authors:** “Fake” image was the first idea that came to us during the manuscript writings. However, we agree that this term is odd. Also, since the image was not “resampled,” but the pixels were resampled, we suggest using the term “pixel-resampling image” instead. We hope this will attend your concerns.
v. Line 201 (normal distribution): have the authors determined (by an appropriate statistical method) if this assumption is correct?

**Authors:** This statement appears in Mainly (2004). We included this reference on line 215 to support our statement better. However, we did not test this assumption. Instead, we checked visually that the D2 was, in fact, following a chi-squared distribution. This information was included in the manuscript (lines 215 and 216) to make it clear to the reader.

vi. Line 217 (R, G, and B): Were the three images (R, G, and B) analyzed individually or was a combination of the 3 used in a single image used?

**Authors:** Initially, in step 1, as stated on line 217 (original manuscript), we analyzed the RGB image, or else, all three bands combined.

vii. Line 218 (NIR): Please elaborate more. What wavelengths (all, solely a few selected wavelengths, etc.)?

**Authors:** In this case, we considered the whole spectra, or else, from 900 to 1700 nm, as presented by the instrument. We included extra information about the NIR instrument and the wavelengths: we used a DLP® NIRscan Nano™ EVM spectrometer (Texas Instruments Inc., Dallas, Texas, USA), with a spectral range of 900-1700 nm and at a 1.32 nm resolution. We presented this information on lines 112 to 115 of the revised manuscript.

viii. Lines 255 and 256 (all bands together (AUC = 0.6289)): Doesn’t the blue line in graph 4A represents “all bands (NIR+RGB) together”? And if so, how can the AUC of that line be so close to the AUC of the R band (black line on 4B)? They seen quite apart analyzing figure 4A and 4B.

**Authors:** Thanks for observing our mistake in not informing that the term “all bands” should be, in fact, “all image bands”, or else, the RGB together. It does not involve NIR in this comparison yet. We have corrected this information in the text (lines 269 and 270).

ix. Line 286 (0.1057 and 0.0579 for classes C2 and C3, respectively): Both these values are higher than 0.05. Doesn’t that the majority of the attributes from individuals from the same family (C2) and from different families (C3) are not statistically different?

**Authors:** It would not be statistically different if we fixed the p-values to 0.05 when stating any conclusion. On the other hand, if we use the p-values to measure the casualty of the results, we prefer to interpret it as a measure of the strength of the comparison towards the null hypothesis of no difference between the two samples. In other words, the higher the p-value, the less different the samples are; the smaller the p-value, the more different the samples are. Important to highlight that we slightly corrected the information presented on lines 298 to 304 observed by the other reviewer. Because of this correction, the p-value of class C3 has decreased from 0.0579 to 0.0294, which makes our discussion in the manuscript even better.

x. Line 302 (identify the p-value threshold that would separate): shouldn’t that be 0.05 (for 95% significance)?

**Authors:** Not necessarily. In many research areas, like the Medical one, researchers might consider using a minimal value for considering a new drug as better than the actual drug. In the agricultural area, researchers could state a larger p-value (5 to 10%) as necessary information for rejecting a null hypothesis in favor of a new fertilizer or variety, for example. Since we are proposing a new protocol to discriminate samples using only one image of each, from lines 301 to 315 of the original manuscript, we briefly discussed this issue. In this way, we believe the reader could better perceive his findings or even start new research on this subject.
