# Supplementary material for: Insights and protocols for discrimination of sugarcane clones by dissimilarity measures on RGB and NIR data
Source: PLoS One. 2023 Jul 20;18(7):e0288508. doi: 10.1371/journal.pone.0288508 (PMC10359001; doi:10.1371/journal.pone.0288508)
Supplement: Attachment — Submitted filename: Response to Reviewers.docx [file pone.0288508.s003.docx]

**Rebuttal letter to the academic editor and reviewers**

We want to thank all for the suggestions, comments, and criticisms regarding our manuscript. The reviewers' contributions will, for sure, improve the manuscript. Below, one can find, in red, the authors’ responses to each question or comment. The highlighted changes were made to the ‘Revised Manuscript with Track Changes’ file.

**Journal Requirements:**

Please review your reference list to ensure that it is complete and correct. If you have cited papers that have been retracted, please include the rationale for doing so in the manuscript text, or remove these references and replace them with relevant current references. Any changes to the reference list should be mentioned in the rebuttal letter accompanying your revised manuscript. If you need to cite a retracted article, indicate the article’s retracted status in the References list and also include a citation and full reference for the retraction notice.

**Authors:** We have reviewed the list of references and have not detected any modifications that should be mentioned.

**Review Comments to the Author:**

*Reviewer #2:*

The authors have addressed all comments raised by the reviewers. There is only one issue that I would suggest to be avoided in the future, that is commit to activities in the future and not in the current work, such as reported here:

i. (Authors: If the objective were to use the NIR in predicting some attribute of agronomic interest (for example, fiber content, sucrose content), the association between wavelengths and organic bonds would be interesting. Our research group is developing future studies involving this type of investigation. However, for the present manuscript, our main objective was to evaluate sample discrimination between individuals only. Therefore, we did not state any discussion regarding that matter.)

and here

ii. (However, as previously mentioned, the primary aim of our research is only to differentiate genotypes and not to associate them with a specific trait. This topic is another front of study under development by our research group. The suggested papers will undoubtedly contribute to future ideas and discussions.)

I do not see the point of why not doing it in the current study, making a strong research and report it. Instead, it seems more that there will be further similar studies to the current one, this splitting the work and its relevance.

**Authors:** We apologize for referring to future work in our response. The present dataset will not be used to infer the association between wavelengths and organic bonds. As we stated before, for the present manuscript, our main objective was to evaluate sample discrimination between individuals only, with no further discussion or inference regarding the inner part of the samples. What we mentioned about future work is still being planned and will be developed by other team members in new experiments planned for this specific purpose.

*Reviewer #3:*

Insights and protocols for discrimination of sugarcane clones by dissimilarity measures on RGB and NIR datafocus on early genetic improvement/selection while considering extensive phenotyping techniques. Perhaps, with this data, RGB/or R images can facilitate the selection. I still have few queries:

i. Fig 3 explains clearly about discrimination of clones based on the Euclidean distance. Exact clones C1 showed lower values of Euclidean distances concentration compared to other. Please explain in detail regarding the change in axis labels in 3A, 3B and 3C.

**Authors:** In Fig. 3, the change in the axis labels in Figs 3A, 3B, and 3C is due to the number of variables involved in calculating the Euclidean distance. In Fig 3A, three variables (R, G, and B) were used; in Fig 3B, only one variable (R); and in Fig 3C, there were 605 variables (corresponding to wavelengths). The different number of variables are causing the changes between the axis labels. We included this information in the text (lines 236 to 240).

ii. Fig 4 suggests that superimposition of NIR (green), and the combination of RGB and NIR (blue line) and author explained that more attributes from NIR spectra (605 wavelengths) leads to overlapping. In such case, can we have a curve of RGB and R together to clear understanding?

**Authors:** Both curves appear in the graphs: the RGB curve in Fig 4A and the R curve in Fig 4B. As the axes are on the same scale, the comparison is immediate, and it can be seen that they are practically identical.

iii. It is agreed that D2 would never be zero and h value should be calculated if researchers want to differentiate ap per D2.Since it has not been done and opined that D2 might be influenced by number of attributes extracted from the images, therefore p-value associated with the D2was referred as an additional measure for decision-making. Although associated with D2 (no significant difference among orange and green line), why doesn't the quantity of attributes affect the p value (significant difference among orange and green line)?

**Authors:** Assuming the same number of attributes, D2 would be considerable if we compared different samples. However, if the samples are very similar, the D2 would be small. The magnitude of D2 is conditioned to the number of attributes used in calculating the statistic. In turn, the p-value that will be associated with the D2 obtained in each case would be obtained from a specific $\chi^{2}$ distribution, since the distribution is defined by its degree of freedom, which depends on the number of variables. If you are referring to Fig 5, we are visually comparing classes C1 and C2. In both graphs, it is verified that both the D2 and the p-value indicate that individuals from the C1 class tend to give lower results for D2 and higher for p-value, which is consistent with our discussion presented in the article.

iv. Please provide citation which also suggests that p value is better than D2 value.

**Authors:** We are not saying that the p-value will replace the D2 or is better than the D2, but that they can be used together to aid the researcher in decision-making (see comments that follow lines 329 of the original manuscript). This procedure appears in books dealing with statistical inference.

v. I would like suggest to choose Kullback-Leibler discrepancy as dissimilarity measure.

**Authors:** Thank you for the suggestion. However, as we are working with the idea of comparing samples two-by-two and not comparing probabilistic distributions, according to the suggested application for the use of KL-divergence in Joyce (2011, https://doi.org/10.1007/978-3-642-04898-2_327), we opted for the measures used in the article.

*Reviewer #4*:

I believe that the revised manuscript may now be considered for publication, though the normal distribution issue is, still, not fully addressed.

**Authors:** Thank you for your consideration.
